# Supplementary material for: Characterization of Sodium Alginate-Based Films Blended with Olive Leaf and Laurel Leaf Extracts Obtained by Ultrasound-Assisted Technology
Source: Foods. 2023 Nov 9;12(22):4076. doi: 10.3390/foods12224076 (PMC10670003; doi:10.3390/foods12224076)
Supplement: Supplementary file 1 [file foods-12-04076-s001.zip › foods-2681280-supplementary.pdf]

Supplementary Table S1. Total phenolic compounds (TPC) on simulant media expressed in mg/L gallic acid equivalent.

Filme	Simulant	1d	3d	6d	9d	15d	*p*
SA + LLE 1%	Water	466.19 ±15.10^{de}					
	Eth. 10%	458.70 ±41.87^{de}					
	Eth. 95%	41.30 ±2.23^{g}	63.69 ±6.86^{b}	48.64 ±6.9^{c}	39.12 ±6.74^{e}	126.70 ±103.67^{c}	n.s.
SA + LLE 2%	Water	1028.47 ±97.92^{a}					
	Eth. 10%	832.80 ±65.07^{b}					
	Eth. 95%	318.48 ±43.56^{ef}	371.91 ±26.79^{a}	429.45 ±128.06^{ab}	436.158 ±78.973^{bc}	471.13 ±80.91^{ab}	n.s.
SA + OLE 1%	Water	365.68 ±39.82^{ef}					
	Eth. 10%	495.90 ±47.23^{e}					
	Eth. 95%	245.23 ±6.04$^{fg\ C}$	421.48 ±19.48^{aA}	336.07 ±24.34$^{b\ B}$	319.15 ±39.39$^{cd\ B}$	348.45 ±33.57$^{bc\ AB}$	***
SA + OLE 2%	Water	666.22 ±65.38^{c}					
	Eth. 10%	565.98 ±45.24^{cd}					
	Eth. 95%	316.95 ±40.42$^{ef\ B}$	461.05 ±70.68$^{a\ AB}$	513.43 ±21.39$^{a\ AB}$	556.70 ±100.79$^{ab\ AB}$	695.15 ±151.11$^{a\ A}$	*
SA + LLE 0.5% + OLE 0.5%	Water	408.45 ±15.25^{de}					
	Eth. 10%	418.26 ±44.4^{de}					
	Eth. 95%	108.14 ±22.90^{g}	133.90 ±23.54^{b}	153.53 ±32.12^{c}	175.03 ±33.83^{de}	181.22 ±42.11^{c}	n.s.
SA + LLE 1% + OLE 1%	Water	876.72 ±17.04^{b}					
	Eth. 10%	898.42 ±94.61^{ab}					
	Eth. 95%	348.53 ±23.87$^{ef\ D}$	449.92 ±15.26$^{a\ C}$	548.53 ±12.69$^{a\ B}$	616.27 ±40.15$^{a\ AB}$	642.71 ±56.86$^{a\ A}$	***
p		***	***	***	***	***	

SA – sodium alginate films; OLE – olive leaf extract; LLE – laurel leaf extract. For storage time (rows), means with small letters differ significantly, *P < 0.5, ***P < 0.001. For films (columns), means with capital letters differ significantly, *P < 0.5, ***P < 0.001. n.s. – non-significant.

Supplementary Table S2. Antioxidant activity in simulant media expressed in mmol Trolox equivalent/L simulant.

Filme	Simulant	1d	3d	6d	9d	15d	*p*
SA + LLE 1%	Water	3.40 ±0.22^{cde}					
	Eth. 10%	3.13 ±0.45^{de}					
	Eth. 95%	0.63 ±0.10$^{g\ A}$	0.64 ±0.12$^{b\ A}$	0.23 ±0.07$^{b\ B}$	0.38 ±0.08$^{b\ AB}$	0.56 ±0.03$^{b\ A}$	**
SA + LLE 2%	Water	8.07 ±1.80^{a}					
	Eth. 10%	5.23 ±0.33^{b}					
	Eth. 95%	3.04 ±0.47$^{def\ B}$	3.41 ±0.75^{a}	3.15 ±0.50^{a}	3.06 ±0.30^{a}	3.61 ±0.38^{a}	n.s.
SA + OLE 1%	Water	1.96 ±0.50^{defg}					
	Eth. 10%	2.47±0.33^{defg}					
	Eth. 95%	1.36 ±0.01$^{fg\ C}$	2.42 ±0.12$^{a\ B}$	1.81 ±0.16$^{cd\ C}$	1.80 ±0.10$^{c\ C}$	3.30 ±0.31$^{a\ A}$	***
SA + OLE 2%	Water	3.51 ±0.36^{bcd}					
	Eth. 10%	2.77±1.13^{def}					
	Eth. 95%	1.79 ±0.22$^{efg\ B}$	2.83 ±0.74$^{a\ AB}$	3.11 ±0.77$^{a\ AB}$	3.35±0.62$^{a\ AB}$	4.20 ±1.12$^{a\ A}$	*
SA + LLE 0.5% + OLE 0.5%	Water	2.69 ±0.28^{def}					
	Eth. 10%	3.04 ±0.37^{def}					
	Eth. 95%	0.59 ±0.15$^{g\ B}$	0.96 ±0.10$^{b\ AB}$	1.19 ±0.11$^{bc\ AB}$	1.58 ±0.47$^{bc\ A}$	1.42 ±0.08$^{b\ A}$	**
SA + LLE 1% + OLE 1%	Water	4.50 ±0.14^{bc}					
	Eth. 10%	5.16 ±0.25^{b}					
	Eth. 95%	2.16 ±0.22$^{defg\ C}$	2.88 ±0.09$^{a\ B}$	2.95 ±0.14$^{ad\ B}$	3.22 ±0.10$^{a\ AB}$	3.40 ±0.13$^{a\ A}$	***
p		***	***	***	***	***	

SA – sodium alginate films; OLE – olive leaf extract; LLE – laurel leaf extract. For storage time (rows), means with small letters differ significantly, *P < 0.5, **P < 0.01, ***P < 0.001. For films (columns), means with capital letters differ significantly, *P < 0.5, **P < 0.01, ***P < 0.001. n.s. – non-significant.
